# Rapid Cycle Deliberate Practice in Virtual Reality: Teaching Transvenous Pacemaker Insertion to Emergency Medicine Residents

**DOI:** 10.7759/cureus.18503

**Published:** 2021-10-05

**Authors:** Cynthia Peng, Kristen M Ng, Kelly N Roszczynialski, Steven J Warrington, Kimberly Schertzer

**Affiliations:** 1 Emergency Medicine, Stanford University School of Medicine, Palo Alto, USA; 2 Emergency Medicine, Edward Via College of Osteopathic Medicine, Blacksburg, USA

**Keywords:** simulation medicine, procedure training, simulation in medical education, education and training of medical students and doctors (specialist and phd)), simulation, virtual reality simulation, high-risk

## Abstract

Introduction

Transvenous pacemaker insertion is a critical life-saving procedure that is infrequently performed. Traditional mannequin-based training paradigms are resource intensive and sometimes inadequate due to time constraints. Rapid Cycle Deliberate Practice (RCDP) is an effective teaching modality for highly scripted procedures. We propose using a simulation-based technique of RCDP in virtual reality (VR) to teach this procedure.

Methods

Sixteen emergency medicine residents were recruited. A pre-survey was administered at the start of the session, followed by a baseline task trainer checklist-based assessment. This checklist was created based on expert consensus. Participants then underwent the RCDP VR intervention with a subsequent repeat checklist-based assessment as well as a post-survey.

Results

Post-test scores were found to be significantly higher than pre-test scores after residents completed VR deliberate practice simulation (19.5±3.5 vs 24.1±2.0; p<0.001). Subanalysis did not reveal any significant difference based on post-graduate year, previous performance of procedure on a live patient, or previous VR experience. The experience increased participant feelings of preparedness and comfort in performing the procedure (2-disagree vs 4-agree) based on a 5-point Likert scale.

Conclusions

Virtual reality using RCDP to teach transvenous pacemaker insertion demonstrated an improvement in task trainer performance. Further investigation into whether this translates into better patient outcomes or can be generalized to other procedures needs to be considered.

## Introduction

Temporary transvenous pacemaker insertion is a critical life-saving procedure that requires a multitude of steps and proficiency in both cognitive and technical skills. Traditionally, mannequin-based simulation is used to train resident physicians in this infrequently performed procedure [1]. However, studies suggest that current training models are not optimal. In one survey, 50% of 80 medical professionals were unsatisfied with their training in this procedure [2]. Another study evaluating the number of attempts of deliberate practice to achieve competency in cardiac pacing (involving both transcutaneous and transvenous pacing) found that the total number of attempts needed to achieve competency was a median of nine attempts, which is greater than the six required by the Accreditation Council of Graduate Medical Education (ACGME) [1]. Inadequate training can lead to critical delays and complications, which in one study was cited in up to 23% of those placed by both cardiologists and emergency faculty physicians [3].

Given the complexity of the procedure, simulation-based mastery learning for this procedure could be optimized by applying deliberate practice to targeted steps. Training in this procedure could be improved by using a technique called Rapid Cycle Deliberate Practice (RCDP). In this approach, rather than an end-of-scenario debrief, the learner receives a series of micro-debriefs throughout the simulation. They are then given the opportunity to repeat and apply the feedback to the items addressed in a ‘pause, debrief, rewind and try again’ model [4]. While RCDP instruction for procedures has been effectively demonstrated in the past, this technique has yet to be reported in transvenous pacemaker placement [4]. One limiting reason may be due to the variable amount of time necessary to achieve mastery for each unique learner. This would result in requiring unpredictable amounts of resources in faculty and trainee time, as well as facility time and space.

A solution to bring this application of RCDP to learning transvenous pacemaker insertion may be the use of virtual reality (VR). VR training has already been shown to be useful in procedural training [5-9]. One study showed that error rates of surgical residents were reduced three-fold during their first ten laparoscopic procedures [8]. The benefit of VR involves the ability to perform repetitive practice with faster resets and objective, unbiased measures consisting of preset checklists [10]. In a competency-based teaching model where time is variable, VR is an attractive modality that may reduce the amount of time and resources necessary to teach this procedure. Traditional teaching in small group settings may evolve into a more personalized paradigm as VR offers a way to scale effective procedural teaching. 

We propose the novel development of a VR experience to teach transvenous pacemaker insertion for emergency medicine resident physicians using the principles of RCDP. Our hypothesis is that teaching pacemaker insertion with VR will show significant improvement in the performance of critical actions in this procedure when compared to pre-testing. Overall, we intend to show that VR is a viable modality for individualized teaching of temporary transvenous pacemaker insertion.

## Materials and methods

Technology development

Simulation is an effective method for teaching procedural skills in emergency medicine [11]. For the low-frequency, high-risk procedures, such as transvenous pacemaker insertion, simulation is key for appropriate practice in a safe environment. The number of steps and unfamiliarity with equipment increases the complexity of the procedure and the risk for error. However, technology represents an instructional strategy that may be more effective at teaching safe and successful completion of the procedure. In accordance with the Mayer Multimedia principle, the usage of immersive learning technologies portends better generative processing for learning [12]. Specifically, with cardiac pacing, technological advances such as developments in software have been shown to improve understanding, management, and confidence level in junior residents [13].

For our experiment, we created a custom case in coordination with a VR healthcare simulation company (www.SimXVR.com). After donning a headset and two hand controllers with triggers (Oculus Quest^TM^), the learner can interact with virtual equipment and mimic hand motions such as gripping, pushing, and twisting. In the virtual environment, the learner can perform the procedural steps. Similar to traditional simulation, an instructor can control advancement of the case and speak with the learner while they are in the case. The focus is to give the learner a cognitive framework for the procedure in the order of operations rather than teach the manual dexterity. One major benefit to using VR for deliberate practice rather than traditional mannequin-based instruction is that a faster initial set-up and reset of equipment allows for more efficient use of time in repetitive practice.

In conjunction with the virtual experience, we developed a procedural checklist for the transvenous pacemaker insertion. The checklist was defined through the expert consensus of a double-boarded critical care emergency medicine physician and a cardiologist, who collectively had greater than thirty insertions on live patients. Each item was defined as critical or highly preferred and subsequently reviewed for the ease of implementation in the virtual environment. Items that were not feasible to implement due to limitations in technology, such as natural language processing features, were modified but remained aligned with the learning objectives.

The checklist was split into a series of five checkpoints: (1) sterilization and preparation for cordis insertion, (2) cordis insertion, (3) preparation of transvenous pacer wire, (4) transvenous pacemaker insertion, and (5) securing of pacemaker. The goal of segmenting the checklist was to decrease the cognitive load on the learner and to help them process the steps in series with smaller amounts of working memory [12]. Successful completion of each critical item list within one checkpoint allowed the learner to progress to the next segment. If items were incorrect or missing, participants were able to view the list of required tasks within the checkpoint and push “retry” to return to the beginning of that segment.

The RCDP VR experience was piloted with five emergency medicine simulation faculty and feedback was provided to the developer for modification. Items on the checklist that were unable to be completed by more than two faculty members due to practice variation were adjusted.

Study design

Our study was a single-center, prospective pilot study approved by the institutional review board. Inclusion criteria for our study included participants who are active emergency medicine resident physicians at our institution. Sixteen voluntary participants were recruited through electronic correspondence and consented for the study.

Facilitators for the study included three simulation fellowship-trained emergency medicine physicians with prior VR and RCDP experience. Prior to the implementation of the study, facilitators were familiarized with the checklist and the mannequin-based assessment. All facilitators of the VR experience were required to go through the virtual reality experience prior to administration.

The participants were provided with a Qualtrics survey to assess their previous experience with VR and the procedure, current comfort and preparedness level, and general attitudes towards the use of VR (Table 2). Participants were then asked to perform the transvenous pacemaker insertion using an Arrow Transvenous Pacemaker kit and single chamber pacemaker on a Laerdal Central Line Man task trainer. They were observed and rated by trained faculty using the 28-item checklist. Prior to VR intervention, each participant viewed a 5-minute orientation video with an introduction on how to interact with the virtual environment. During the VR experience, a facilitator monitored the participant's progress via a wifi-connected computer, while answering pointed technical questions and ensuring checkpoint advancement. Immediately following the VR intervention, another task trainer-based assessment was performed and rated. Finally, all resident physicians who underwent the VR experience were asked to complete a post-experience survey. 

The data were assessed for normality using descriptive statistics and the Shapiro-Wilk test. Differences in checklist scores were analyzed using parametric, paired t-test and the sub-analysis was performed using the Mann-Whitney U test. Descriptive analysis was used to interpret the 5-point Likert survey results. All statistical analyses were performed using SPSSv27. 

## Results

Participants were represented across all post-graduate years (PGY) one through four with the majority (38%) representing the PGY4 cohort (Table 1).

**Table 1:** Demographic characteristics of participants. *Incomplete entries for this category were excluded from the analysis.

Characteristic	Frequency
Age*	
< or =30 years old	10 (56%)
>30 years old	8 (44%)
Training year	
PGY2	9 (45%)
PGY3	11 (55%)
Gender*	
Female	6 (32%)
Male	13 (68%)
Ethnicity	
Caucasian	15 (75%)
Other	5 (25%)
Family status*	
Single	7 (37%)
Married or domestic partnership	12 (63%)
Children	4 (21%)

The participant sample was predominantly male (69%). Slightly less than half (44%) of participants had experience with virtual reality in the past. 69% of participants had performed the transvenous pacemaker insertion on an actual patient compared to 56% having performed this on a mannequin. Over half (63%) had viewed an online demonstration of the procedure prior to the investigation.

**Figure 1:**
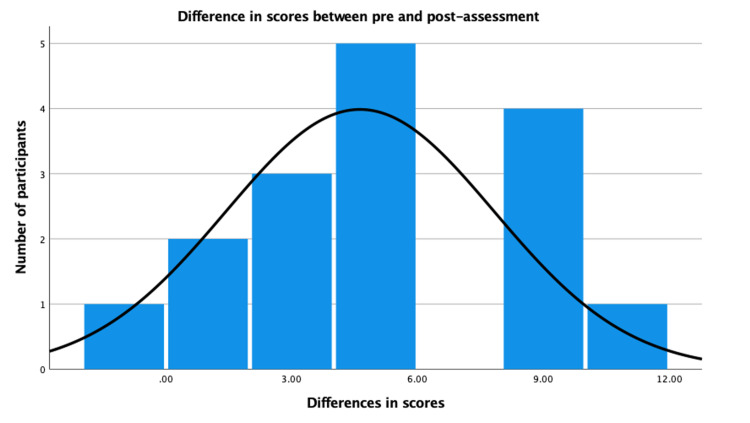
Graphical representation of observed differences between pre and post-assessment scores of all participants.

The post-test scores were found to be significantly higher in participants after experiencing the VR simulation (19.5±3.5 vs 24.1±2.0; p<0.001) with a mean improvement of 4.6±4.0 (Figure 1). 

Participants who had performed this procedure previously on an actual patient did not have any significant difference in the pre-test compared to those without that experience (18.9±4.2 vs 20.1±2.9). Sub-analysis also revealed no difference in mean pre-test scores or differences in improvement of scores between juniors and seniors (19.3±2.4 vs 19.6±3.5, 4.0±3.9 vs 4.6±3.2). Prior VR experience also did not impact improvement on performance (4.7±3.5 vs 4.6±3.0). There were no significant differences in the improvement of scores based on gender (3.8±2.8 vs 6.4±3.6). 

Overall, participant perceptions towards VR use in medical education were more positive after the experience (from 4-agree to 5-strongly agree). Feelings of comfort and preparedness to do the procedure also dramatically increased (2-disagree to 4-agree) (Table 2). 

**Table 2:** 5-point Likert scale median response levels on pre and post-experience survey regarding attitudes and preparedness.

	Pre	Post
How immersive would you say your VR experience was?	3	5
Do you feel VR should be used to teach in medical education?	4	5
Do you feel VR should be used to teach procedures?	4	5
How comfortable do you currently feel performing this procedure in a live patient?	2	4
How prepared do you currently feel performing this procedure in a live patient?	2	4

## Discussion

We have found that residents who were engaged in this RCDP VR experience demonstrated significantly better procedural competency in transvenous pacemaker insertion than their initial pre-test performance. Correspondingly, residents experienced increased comfort and feelings of preparedness with the procedure as well as a more positive attitude towards virtual reality use in medical education and procedural teaching. Here, we demonstrate how core components of RCDP can be successfully incorporated into a VR simulation case.

Our study underscores the indiscriminate potential of VR to teach medical learners. Prior experience in VR was not a necessary requirement to learning from the virtual environment and enhancing performance on the post-assessment. This highlights the ability of the current generation of learners to quickly adapt and utilize various platforms for medical education. In addition, certain participant characteristics, such as gender, did not appear to significantly impact performance. This is slightly contrary to some studies that have shown females to perform better in acquisition of suturing skills using VR [14]. Our pilot study, however, was not powered to detect this difference and further investigation is needed.

With new requirements as a result of a global pandemic and increasingly networked society, medical education is turning towards virtual and small group options to teach learners critical procedures [15]. VR offers the ability to remotely operationalize individualized training without requiring significant physical resources. In our study, for example, a 10 ft by 10 ft open space for the Oculus system in a wifi-configured area is the extent of the physical space required. Restructuring training around VR could decrease faculty time commitment and create more opportunities for learners to engage and practice these critical procedures [10]. By engaging in a virtual network, all levels of emergency physicians from resident physicians to seasoned faculty could practice high acuity, low frequency procedures such as transvenous pacemaker insertion on a more regular basis.

It is important also to note the potential return of investment (ROI) and feasibility of adding a virtual training component to procedural training in residencies. Currently, there is a paucity of literature on such topics. In addition, costs associated with simulation-based medical education in general, and VR are not static. Nevertheless, VR training has been associated with decreased operating times and error rates in laparoscopic surgery [8,16,17]. We found that the tangible benefit to patient care, increase in learner confidence, and reduction in cost of equipment, space, and faculty time to utilize this training tool, are compelling potential returns of investment.

Limitations

While the results of this pilot study are promising, various limitations do exist. First, our cohort was a convenience sample at a single center within one specialty. Our sample size also prevented adequate powering to detect differences between certain subgroups. Our sample was skewed towards more senior residents and male participants, which may not be representative of a broader population. The use of an objective measure of checklist items provides some additional standardization with regards to assessment, but still does not allow speculation about the translation of virtual reality procedural skills to patient care, where factors like anatomic variation and bleeding may provide obstacles to successful execution of the procedure. 

## Conclusions

Our intervention demonstrated that RCDP using virtual reality to teach transvenous pacemaker placement may be a viable modality. As technology continues to develop, new capabilities such as haptic feedback or integration of augmented reality (AR), an overlay of a virtual image over a physically present task trainer, may allow for increased tactile practice and feedback. In addition, increased familiarity with VR and natural language processing may make it possible to create an autonomous VR learning experience for individuals to pursue deliberate practice asynchronously without instructor involvement. Future areas of study may also include evaluating patient outcomes in association with skills trained using the virtual environment as well as application of this technology to other procedures and disciplines.
